# Fiat Justitia, Etc.

**Published:** 1883-01

**Authors:** 


### FIAT JUSTITIA—RUAT CŒLUM.

A subscriber writes: "I am glad you give us country doctors faithful criticisms of new publications. I have been taken in on several occasions by buying books advertised and indorsed by homœopathic journals. After buying, found them—*no good.*"

